# Therapist-Facilitated Virtual Reality Intervention for Social Anxiety Among Youth: Protocol for a Randomized Controlled Trial

**DOI:** 10.2196/86937

**Published:** 2026-08-11

**Authors:** Yi Zhou Wang, Ming Tak Hue, Kung Wong Lau, De Hui Zhou, Siu Man Ng, Xue Weng

**Affiliations:** 1 Department of Counselling and Psychology, Hong Kong Shue Yan University, 8 Wai Cui Cresent, Hong Kong, China (Hong Kong), +852 2570 7110; 2 Department of Applied Data Science, Hong Kong Shue Yan University, Hong Kong, China (Hong Kong); 3 Department of Social Work and Social Administration, University of Hong Kong, Hong Kong, China (Hong Kong); 4 Institute of Advanced Studies in Humanities and Social Sciences, Beijing Normal University, Zhuhai, China

**Keywords:** digital health, social anxiety, virtual reality, randomized controlled trial, youth

## Abstract

**Background:**

Youth social anxiety is rising; however, evidence for therapist-guided virtual reality (VR) exposure remains sparse.

**Objective:**

This randomized controlled trial will test whether such a VR program is associated with reductions in social-anxiety symptoms in youth aged 15 to 25 years.

**Methods:**

Participants will be recruited from schools, tertiary institutions, and community centers in Hong Kong and screened for eligibility. Youth aged 15 to 25 years who score 25 or higher on the Social Phobia Inventory (SPIN) will be randomly allocated to either the VR intervention group or the waitlist control group in a 1:1 ratio. The VR group will receive a 6-session therapist-facilitated VR intervention, focusing on exposure to realistic social scenarios, real-time physiological monitoring, and AI-driven adaptive support. The control group will not receive the VR intervention during the primary assessment period but will be offered access after the 3-month follow-up assessment. Both groups will be assessed at baseline, 1 month (postintervention), and 3 months for the primary outcome of social anxiety (SPIN) and the secondary outcome of depression (Patient Health Questionnaire-9). An exploratory cost analysis will estimate the cost per unit reduction in SPIN scores.

**Results:**

A total of 90 participants (45 in each group) will be recruited. Participant enrollment began in March 2026, and data collection will continue until the required sample size is reached. The primary findings are expected to be published in late 2027. Outcome analyses will use a linear mixed effects model under the intention-to-treat principle, with missing data accounted for under the assumption of missing-at-random.

**Conclusions:**

This trial will provide preliminary evidence on the efficacy, feasibility, and acceptability of a therapist-facilitated VR intervention for youth social anxiety in Hong Kong. Given the waitlist comparator and 3-month follow-up period, findings will not establish VR-specific effects, long-term effectiveness, or implementation-level cost-effectiveness.

## Introduction

### Social Anxiety Among Youth

Social anxiety, also known as social phobia, is characterized by a marked fear of social situations where individuals may be scrutinized by others, often leading to avoidance behaviors and distress [1,2]. It, often arising in adolescence, is among the most common anxiety disorders affecting youth and can substantially disrupt academic performance, peer relationships, and emotional well-being [3,4]. A central feature of social anxiety is extreme sensitivity to negative evaluation, perpetuating cycles of self-doubt and anticipatory anxiety [4]. Among young people, persistent concerns over potential judgment can lead to social withdrawal and diminished social support, thereby exacerbating loneliness and isolation [5]. Indeed, in an investigation involving adolescents and young adults, researchers found that social anxiety often manifests before major depression, underscoring the urgent need for early screening and targeted interventions in this population [6].

### Therapist-Facilitated Virtual Reality Interventions

Virtual reality (VR)–based interventions provide immersive and controllable environments in which anxiety-provoking social or performance situations can be simulated [7,8]. For youth with social anxiety, VR may be particularly useful because feared situations can be presented gradually, repeated safely, and adjusted according to the participant’s level of difficulty; however, youth-specific evidence remains limited and requires further controlled evaluation [9]. Compared with in vivo exposure alone, VR may reduce some practical barriers by allowing exposure tasks to be delivered in a private and standardized setting, although transfer to real-world functioning should be examined empirically rather than assumed [7,8,10]. Therapist facilitation may further strengthen VR-based interventions. A trained therapist can help participants identify feared predictions and safety behaviors, prepare for exposure tasks, monitor distress, and consolidate learning through debriefing and homework planning [11,12]. However, evidence on therapist-facilitated VR interventions for youth social anxiety remains limited, and the extent to which symptom improvement transfers to daily functioning requires further empirical investigation [9,10].

### Research Gaps

Robust evidence on VR treatment for adolescent social anxiety is still sparse, particularly from randomized controlled trials (RCTs). This gap is noteworthy because adolescents, often described as “digital natives,” are generally receptive to technology-mediated care—a factor that can boost both engagement and adherence [9]. Although a small body of research indicates that self-guided VR programs can reduce anxiety by providing convenient, on-demand, and relatively low-cost exposure practice [7,8], the added value of therapist-facilitated VR remains largely untested. In a guided format, clinicians can give real-time feedback, fine-tune exposure tasks, and address comorbid issues such as cognitive distortions or emotional dysregulation as they emerge [13]. This active professional involvement is likely to improve treatment adherence, deepen therapeutic engagement, and optimize clinical outcomes by keeping the exposure simultaneously challenging and safely supported [13].

Additionally, the lack of economic evaluations for VR-based psychological interventions is a critical issue, particularly during times of global economic downturns, when funding for mental health support and research often declines [14]. Without rigorous cost-effectiveness analyses, policymakers may face challenges in allocating resources efficiently, potentially limiting access to innovative digital interventions that could provide scalable, engaging, and sustainable mental health support for young individuals. Addressing these gaps is important for understanding whether VR interventions can be both potentially useful and feasible for broader use in youth mental health care [15].

### Research Objectives and Hypotheses

Our aim is to test the preliminary efficacy of a therapist-facilitated VR intervention for reducing social anxiety symptoms among young individuals, compared with a waitlist control condition. The primary research question is: “Does the therapist-facilitated VR intervention, when compared to waitlist group, result in a reduction of social anxiety symptoms among youths at 1-month (post-treatment) and 3-month follow-up?” Additionally, the study aims to investigate the cost-effectiveness of this intervention by determining “What is the cost per unit reduction in the SPIN score (if applicable)?” The study does not aim to establish implementation effectiveness in routine service settings or definitive generalization to real-world behavior. Our hypotheses are as follows:

- H1: Compared with the waitlist control, a therapist-facilitated VR intervention will reduce social anxiety symptoms at the 1-month (posttreatment) follow-up.
- H2: Compared with the waitlist control, a therapist-facilitated VR intervention will reduce social anxiety symptoms at the 3-month follow-up.
- H3: The VR intervention will demonstrate a cost per unit reduction in social anxiety symptoms, providing an estimate of its economic feasibility.

## Methods

### Trial Design

**Figure 1.**
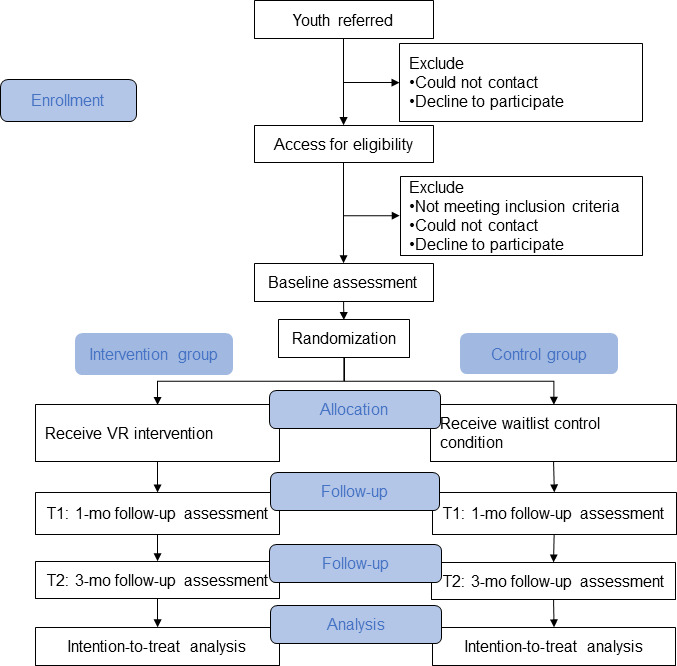
CONSORT (Consolidated Standards of Reporting Trials) flow diagram of the proposed randomized controlled trial. VR: virtual reality.

The design is a 2-arm, statistician-blind, parallel-group RCT to test whether the VR intervention, compared with a waitlist control condition, leads to a reduction in social anxiety among youth. Assessments will be carried out at 0 (baseline, T0), 1 (posttreatment, T1), and 3 (follow-up, T2) months. A summary of the trial design can be seen in Figure 1.

### Ethical Considerations

The protocol and the informed consent form received approval from the Hong Kong Shue Yan University Human Research Ethics Committee (HREC 24-05(M3)). Eligible participants willing to join the study will sign an online consent form. For participants under 18 years of age, additional parental or guardian consent will be obtained. To reduce the likelihood or detection of harm, various safety and security procedures will be implemented, such as excluding potentially psychologically ill participants during the screening stage.

To ensure the confidentiality and security of participant data, all collected information is securely encrypted during transmission and storage, preventing unauthorized access. Access to participant data is strictly limited to authorized research personnel. Additionally, personally identifiable information is anonymized or deidentified wherever possible to safeguard participant privacy. Each eligible participant will receive compensation of approximately HK $500 (HK $1=US $0.128 as of July 15, 2026) for their participation in the study.

### Randomization, Allocation Concealment, and Blinding

Participants will be randomized after the baseline assessment using computer-generated block randomization with a 1:1 allocation ratio within pre-established blocks of 90 participants. To ensure balance, a randomized blocking schema with block sizes of 2, 4, or 6 will be implemented. An independent research assistant, uninvolved in statistical analysis, will conduct the randomization. Allocation concealment will be maintained until the participant has completed eligibility screening, provided informed consent, and undergone baseline assessment. The allocation sequence will be accessible only to the independent staff member responsible for randomization. Research staff involved in recruitment and baseline assessment will not have access to the allocation sequence before assignment. Because of the nature of the VR intervention and the waitlist control condition, participants and therapists cannot be blinded after allocation. However, the statistician conducting the primary analysis will remain blinded to group labels until the primary analysis has been completed. Group labels will be coded as group A and group B during data cleaning and primary analysis to reduce analytic bias.

### Recruitment

The trial will enroll 90 participants, allocating 45 to each arm. Allowing for 15% attrition, this sample provides 90% power to detect a treatment effect at T1, assuming an independent samples 2-tailed *t* test, an *α* level of .05, and Cohen *d* of 0.75. The target size also accords with comparable 2-arm VR intervention studies [16].

The first participant was enrolled in March 2026. The study will recruit participants from various settings in Hong Kong, including middle schools, tertiary institutions, and community centers. Adequate participant enrollment will be facilitated through partnerships with collaborating institutions, which will post digital flyers and promotional videos on their social-media platforms to reach the target population. The participants’ inclusion criteria were as follows: youth aged 15 to 25 years with a score of 25 or higher on the Social Phobia Inventory (SPIN). The exclusion criteria were as follows: (1) inability to attempt a baseline assessment (eg, due to being unpermitted to leave a psychiatric ward); (2) photosensitive epilepsy or significant visual, auditory, or balance impairment; (3) currently receiving another intensive psychological intervention; (4) a history of neurological conditions (eg, traumatic brain injury); and (5) insufficient language proficiency to understand the intervention requirements. In this trial, any endorsement of suicidal ideation will trigger crisis intervention procedures. Participants will be promptly flagged, and a trained clinician will notify the school or guardian to ensure appropriate safety measures are taken. School tutors or social workers familiar with students’ mental health will help identify participants unsuitable for the study, especially those whose conditions may be masked in self-report questionnaires. Participants will be recruited and screened online or offline, but will complete all interventions offline. Participants will be briefed through tutorial video clips demonstrating the research flow regarding intervention procedures, timing, and frequency.

### Protocol Development

#### Intervention Group: Therapist-Facilitated VR Treatment

The objective of the VR intervention is to facilitate the relearning of safety in social events among young individuals. The VR intervention involves the implementation of repeated behavioral experiment tests, which aim to assist participants in realizing that their level of safety is higher than their initial perception. All intervention sessions will be delivered by credentialed mental health professionals who meet the following criteria: (1) each therapist will have completed systematic training in cognitive behavioral therapy (CBT) and will have used CBT as their primary clinical modality for a minimum of 1 year; (2) all therapists will hold a master’s degree in clinical psychology, counseling psychology, or social work and maintain an active professional license or registration in their respective jurisdiction; (3) a registered clinical supervisor, possessing at least 8 years of postqualification experience, will oversee the entire treatment process, provide weekly case consultations, and conduct random session audits to ensure fidelity; and (4) an intervention manual will be developed to standardize session structure, therapeutic techniques, and VR scenario use; therapists will complete manual-based training and must demonstrate competency in role-play assessments before treating study participants. The therapists will take charge of the following 3 key phases of the intervention process:

- Intake interview: the mental health worker will assess dysfunctional assumptions and safety behaviors to identify each participant’s key cognitive patterns, allowing VR exposure and cognitive restructuring to be tailored to their most relevant fears and defenses.
- Document establishment: the mental health professional will establish documentation for each participant, which serves to enhance the efficacy of subsequent VR sessions by allowing the mental health professional to better prepare for participants’ responses and provide more targeted guidance. Furthermore, this documentation will also serve as a means to track and record participants’ progress throughout the trial.
- VR session preparation: the mental health professional will assist participants in preparing for the VR sessions. This involves guiding individuals through the intervention process, which includes helping them recognize and address dysfunctional thinking styles, encouraging them to put aside maladaptive defense strategies, conducting debriefing sessions at the conclusion of each session, and providing feedback to tailor the progression of each participant. The mental health professional will also encourage participants to apply the learning from VR into the real world through homework tasks to be carried out between sessions.

#### Theoretical Framework

**Figure 2.**
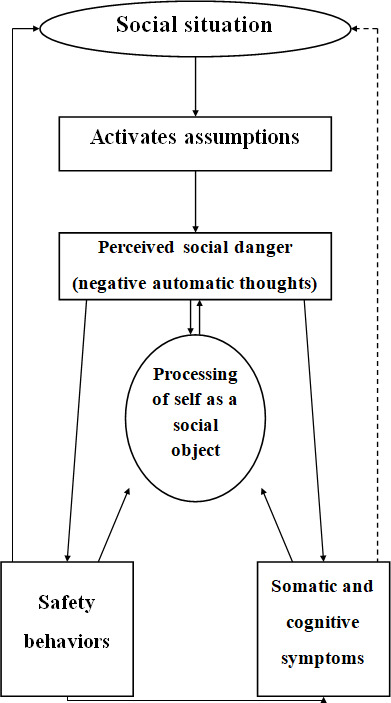
Cognitive model of social anxiety disorder used in this study.

This research will use Clark and Wells’s cognitive model of social phobia (Figure 2) [11]. This model identifies the following 4 key processes that maintain social avoidance [12]:

- Social situation: for adolescents, commonplace events such as giving a short class presentation constitute highly salient “social situations” that can trigger the Clark-and-Wells cycle; epidemiological work shows that such youth-relevant contexts frequently precipitate the first onset of social anxiety [17].
- Activates assumptions: exposure to these situations activates developmentally tuned self-rules. Assumptions such as “If I look awkward, my peers will screenshot and mock me” are shaped by heightened peer sensitivity during adolescence. These maladaptive assumptions prime threat detection systems and lower the threshold for anxiety activation [18].
- Perceived social danger (negative automatic thoughts): the activated rules generate rapid thoughts such as “My hands are shaking—everyone will notice,” inflating both the probability and the cost of negative peer evaluation [19].
- Processing self as a social object: attention turns inward; the adolescent continuously monitors facial redness, voice quiver, or online “likes,” embodying the self as an observable object. This self-focused processing amplifies somatic and cognitive symptoms (eg, tachycardia, blanking) while prompting safety behaviors, such as looking down, muting the mic, and overediting photos, that temporarily soothe anxiety yet block disconfirmation of catastrophic beliefs [20].

#### Intervention Design and VR Scenarios

The intervention comprises 6 sessions of 60 minutes each. This structure could be effective and practical for the target demographic in Hong Kong, considering the efficacy of brief interventions, challenges with longer program retention, and the need for adaptability to the local context [21]. There are 6 VR scenarios: a café, classroom, interview room, public bus, social gathering, and family gathering. Each scenario consists of 10 sequentially structured tasks, increasing in difficulty over time. For example, in a job interview scenario, task 1 involves the participant interacting with a friendly interviewer. Task 2 introduces a neutral, more serious interviewer. Task 3 features a foreign language–speaking interviewer, requiring the participant to respond in a nonnative language (eg, English). Before each task, participants will see an automated dashboard through the VR headset, providing background information and clear instructions on required actions, such as introducing themselves by saying, “Hello, my name is Chan Da Men.” To enhance engagement and support, the system monitors response time and, if no response is detected within 20 seconds, automatically displays assistance options, including taking a short break, requesting support from a psychologist, or extending response time. This structured approach ensures that participants receive gradual exposure, real-time guidance, and adaptive support, enhancing learning, confidence, and anxiety management in the VR intervention.

The ecological validity of the scenarios was strengthened through pilot interviews with youth who reported elevated social anxiety symptoms, as well as consultations with local mental health professionals and a parent (see the “Pilot Study” section for more details). The selected scenarios were chosen because they reflect common social and performance-based contexts encountered by youth in Hong Kong, including classroom participation, public speaking, job or school interviews, commuting and public transport interactions, peer gatherings, café or casual social encounters, and family gatherings. These situations were considered developmentally relevant because adolescents and young adults in Hong Kong often face intense academic expectations, bilingual or multilingual communication demands, dense public social environments, and frequent evaluation in school, university, and early-career settings. The VR tasks within each scenario were therefore designed to reflect locally recognizable interpersonal demands rather than generic exposure tasks. Examples include responding to questions from teachers or interviewers, initiating or maintaining conversations with peers, managing attention from others in public spaces, and coping with perceived evaluation during group or family interactions. This culturally and contextually informed design aims to improve the relevance and acceptability of the intervention for Hong Kong youth while maintaining a standardized structure for trial delivery.

#### Intervention Flow

##### Structure of Each Intervention Session

**Figure 3.**
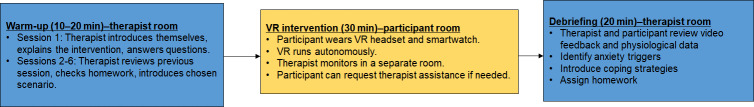
Intervention flowchart of the virtual reality (VR) program.

Before each of the 6 intervention sessions, participants can freely choose from the 6 available VR scenarios based on their personal preferences and therapeutic needs. Each session is structured into 3 key phases (intervention flowchart; Figure 3):

- Warm-up (10‐20 min): in the first session, therapists introduce themselves, provide an overview of the 6-session intervention, explain procedures and safety precautions, and address participants’ questions and concerns to build rapport. From the second to the sixth session, therapists review the chosen scenario, reflect on the previous session, and conduct a homework check-in to reinforce learning and progress.
- VR intervention (30 min): participants move to a separate VR-equipped room, where they wear a VR headset and a smartwatch to track physiological indicators, while a camera records real-time responses for later video feedback analysis. During the 30-minute session, the intervention runs autonomously without therapist assistance. Therapists remain in a monitoring room, receiving real-time feedback on participants’ body movements, voice volume, reaction speed, and fluency, as well as physiological indicators (eg, heart rate and skin conductance). If needed, therapists are on standby to enter the participant’s room on request.
- Debriefing (20 min): after the VR session, participants and therapists review the experience using video feedback and physiological data, identifying specific challenges, such as self-focused attention or cognitive distortions contributing to social anxiety. Therapists introduce coping strategies, such as deep breathing, to help manage anxiety in real-world situations. Before concluding the session, participants receive simple homework assignments, such as practicing breathing exercises in daily social interactions to reinforce learning and real-life application.

##### Intervention Feature 1: Wearable Watch for Real-Time Biological Data Collection

To enhance real-time monitoring of participants’ mental states during the intervention, a commercially available wearable health-monitoring device will be used to collect physiological indicators, including: (1) electrodermal activity (EDA), which reflects changes in skin conductance and indicates levels of physiological arousal and emotional responses; and (2) heart rate variability (HRV), which measures variations in heartbeats associated with stress and emotional regulation. These physiological markers offer objective indicators of anxiety and emotional responses during VR-based exposure tasks.

The collected data will be transmitted in real-time to the back-end control panel (CP), allowing the research team to monitor participants’ anxiety responses during different VR scenarios. If the system detects elevated physiological responses (eg, increased EDA or decreased HRV) during a specific VR task, it signals that the participant may be experiencing heightened anxiety. The biological data will be reviewed postsession with the counseling psychologist. During follow-up discussions, the CP will explore the participant’s experience, validate their emotional responses, and help them develop personalized coping strategies based on their reactions.

##### Intervention Feature 2: AI-Driven Adaptive Support and Ethical Safeguards

During sessions, AI-driven interactive support was provided through Doubao (developed by ByteDance), an AI-powered large language model used in this study primarily for speech recognition and response detection. Based on predefined indicators such as task progression, response delay, and physiological arousal patterns, the system may provide brief and standardized prompts. If signs of frustration, distress, elevated arousal, or prolonged nonresponse are detected, the AI adjusts its approach by offering reassurance, motivational feedback, or brief reminders to use breathing or grounding strategies introduced by the therapist. The AI component is used as a lightweight supportive function; it does not provide diagnoses, make clinical decisions, assess risk independently, or replace therapist judgment. Moreover, after each VR session, Doubao AI generates a personalized postsession report summarizing the participant’s emotional and physiological responses, as well as task completion. The report highlights moments of peak anxiety and recommendations for the next session, including areas for improvement and potential adjustments to intervention difficulty or pacing. Any AI-generated feedback used in debriefing will be reviewed by the therapist, who will decide whether and how to incorporate it into the session discussion. Prompts and feedback templates will be reviewed by the research team to ensure that they are clinically appropriate, nonjudgmental, and consistent with the intervention manual. The back-end code and implementation details can be made available upon reasonable request.

To protect data privacy, identifiable participant information will not be included in AI-generated summaries. Study data will be coded using participant IDs, and access to identifiable data will be restricted to authorized research personnel. Data transmission and storage will follow the approved data security procedures described in the ethics application. Participants will be informed during the consent process that AI-supported feedback may be used as part of the intervention, the type of data involved, the purpose of the AI-generated feedback, and the limits of AI involvement.

### Potential Adverse Effects and Safety Procedures

Potential adverse effects related to VR exposure will be monitored throughout the intervention. These may include cybersickness symptoms such as dizziness, nausea, eyestrain, headache, or disorientation; temporary dissociative-like discomfort or a reduced sense of presence after leaving the immersive environment; and excessive emotional activation during anxiety-provoking social tasks. Before the first VR session, participants will be informed of these possible reactions and reminded that they may pause or stop the session at any time without penalty.

During each VR session, participants will be monitored through real-time behavioral observation, voice responses, task progression, and physiological indicators. Therapists will remain available to enter the VR room or provide support if the participant requests assistance or if the monitoring data suggest substantial distress. Sessions will be paused or terminated if the participant requests to stop, reports significant discomfort, shows signs of cybersickness, becomes unable to continue the task, or if the therapist judges that continuation may not be clinically appropriate.

After each session, therapists will conduct a debriefing to review the participant’s subjective experience, emotional responses, and any adverse reactions. Any adverse events will be documented by the research team and reviewed during supervision. Participants requiring additional psychological support will be referred to appropriate school, community, or clinical services.

### Pilot Study

A pilot study, conducted from January 2025 to April 2025, interviewed 26 individuals with SPIN scores of 25 or higher in Hong Kong to identify the most anxiety-provoking social situations and inform VR scenario design. Participants were asked: “What social settings do you fear the most, such as public speaking or interviews?” Based on their responses, the top 6 most anxiety-inducing situations were identified, including public speaking and job interviews. These findings will guide the development of VR scenarios that replicate realistic high-anxiety situations, ensuring the intervention effectively targets key challenges faced by individuals with social anxiety. Further details of the pilot study will be provided in a forthcoming article titled “Ecological Drivers of Youth Social Anxiety and Stakeholder Perspectives on Virtual Reality Intervention Design: A Qualitative Study in Hong Kong and Mainland China”.

To obtain a broader ecosystem perspective, interviews were conducted with 4 mental health professionals and 1 parent, eliciting views on adolescent social anxiety and their day-to-day challenges. The semistructured guide elicited participants’ views on adolescent social anxiety across five domains: (1) the current prevalence and presentation of the problem, (2) perceived etiological factors, (3) existing supports and resources, (4) gaps or areas in need of improvement, and (5) opinions on whether, and how, VR technology could assist youth with social anxiety.

According to the pilot results, the VR intervention was designed to address the unique characteristics of youth with social anxiety and incorporate suggestions from local mental health professionals. As a result, it stands out from existing VR interventions with 5 key innovative features:

- Targeted focus on social phobia: unlike general VR exposure therapy, this VR intervention is specifically designed to address social anxiety, providing immersive scenarios tailored to gradually expose participants to anxiety-inducing social situations.
- Autonomous VR sessions: the system operates without requiring a therapist to be physically present. If no response is detected for 30 seconds, a prompt appears asking the participant whether they need a break or would like to invite a psychologist into the session for real-time support.
- AI-integrated feedback system: equipped with Doubao AI, the system provides real-time prompts and encouragement based on physiological and behavioral data. For instance, if an elevated heart rate is detected, the AI may prompt: “You’re doing great! Remember the deep breathing exercise from last time? Let’s try it together—inhale for 3 seconds...exhale.”
- Wearable device integration: participants wear a smartwatch that tracks EDA and HRV during VR sessions. These physiological responses are monitored in real time and displayed on the back-end CP, allowing therapists or researchers to assess stress levels remotely.
- Gamification and reward system: this VR intervention includes a point-based system where participants earn experience points for completing tasks. These points can be exchanged for rewards, such as customizing their avatar’s appearance in the virtual world, enhancing engagement and motivation.

### Control Group: Waitlist Control

Participants in the waitlist control group will have access to the VR intervention withheld until after the 3-month follow-up assessment. To prevent confounding effects from relevant concomitant care and interventions, participants will be required to refrain from receiving any other treatments or interventions specifically targeting social phobia during the study period. The rationale for this comparator is that this study is an early-stage RCT designed to estimate the preliminary efficacy, acceptability, feasibility, and safety of a newly developed therapist-facilitated VR intervention in a youth population in Hong Kong. Before conducting a more complex and resource-intensive active-controlled trial, it is necessary to first examine whether the intervention can be delivered as planned, whether participants can tolerate and engage with immersive exposure, and whether the study procedures, assessment schedule, and outcome measures are feasible. The waitlist design will also provide initial estimates of recruitment feasibility, adherence, dropout, outcome variability, and potential treatment signals, which can inform the design and sample size calculation of future trials. Providing delayed access also helps address ethical concerns by allowing control participants to receive the intervention after the primary assessment period.

### Outcomes

**Figure 4.**
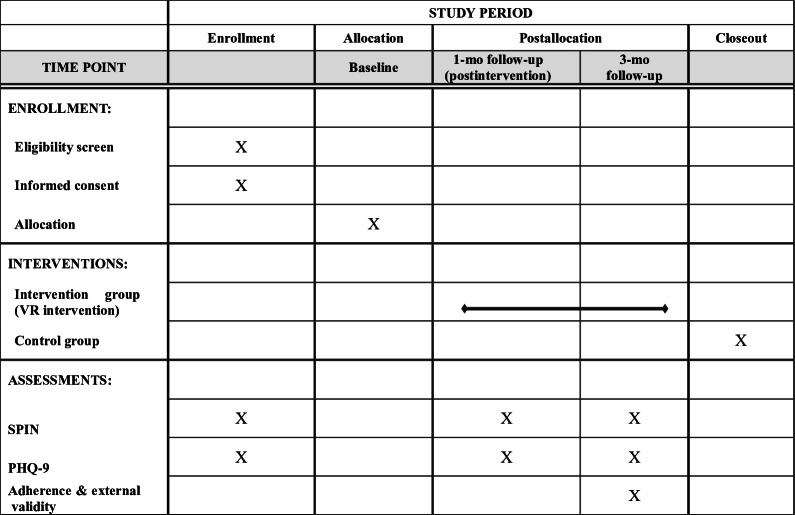
SPIRIT (Standard Protocol Items: Recommendations for Interventional Trials) schedule throughout the study period. PHQ-9: Patient Health Questionnaire-9; SPIN: Social Phobia Inventory; VR: virtual reality.

Basic demographic and clinical data, including age, gender, region, and socioeconomic status, will be collected. The primary outcome will be the SPIN, while the secondary outcome will be the Patient Health Questionnaire-9 (PHQ-9) for depressive symptoms. All outcomes will be self-assessed using the following online measurements at baseline, 1-month (postintervention), and 3-month follow-up (Figure 4).

#### Primary Outcomes: SPIN

Developed by Connor et al [22], SPIN is a brief self-report tool consisting of 17 items that assess fear, avoidance, and physiological discomfort in social situations. It is specifically designed for use with social anxiety disorder [23]. The Chinese version of the SPIN has been examined with good internal consistency (Cronbach *α*=0.85) and test-retest reliability (*r*=0.73). A cutoff value of 25 or higher has demonstrated strong reliability and validity and is commonly used to screen for social anxiety in the Chinese population [24].

#### Secondary Outcomes: PHQ-9

The PHQ-9 is widely used to screen for the presence and severity of depression, monitor the severity of depression over time, and assist with the diagnosis of depression. Each of the 9 items is scored from 0 (“not at all”) to 3 (“nearly every day”), with a total score ranging from 0 to 27. Higher scores indicate more severe depressive symptoms. PHQ-9 scores of 5, 10, 15, and 20 represent mild, moderate, moderately severe, and severe depression, respectively. The Chinese version of the PHQ-9 demonstrates high internal validity and consistency, as evidenced by a Cronbach α of 0.86 and strong test-retest reliability with a correlation coefficient of 0.86, affirming its efficacy for use in Chinese-speaking populations [25].

#### Adherence and Perceived Real-World Application

To assess participant adherence, a fidelity rating scale will be used, specifically measuring key aspects, such as session attendance and completion of assigned homework. Real-world application is a critical issue in evaluating intervention efficacy. To explore perceived real-world application, a mixed methods approach was used. A custom self-report questionnaire was administered at posttreatment and follow-up to measure perceived changes in social functioning, academic or occupational engagement, and real-life application of coping strategies. Items assessed outcomes, such as reduced avoidance, greater participation in daily tasks, and satisfaction with the intervention’s relevance to everyday life. To complement these data, semistructured interviews explored how participants applied therapeutic gains in real-world contexts, including specific examples of social interactions, academic challenges, and emotional regulation.

#### Physiological Data

HRV and EDA data collected during VR sessions will be analyzed using repeated-measures ANOVA to examine within-subject changes across sessions and between-group differences over time. For EDA, any fluctuation exceeding 2 SDs from the participant’s baseline, sustained within a 5-second window before and after the peak, will be flagged as an arousal event. These events will be quantified per session and used as indicators of physiological reactivity. Their frequency and timing will be explored in relation to session content and subjective distress and may serve as predictors or correlates of treatment engagement and emotional processing.

### Statistical Analysis

#### Primary Analysis

The primary analysis will follow the intention-to-treat principle, meaning that all randomized participants will be analyzed according to their assigned groups regardless of intervention adherence, withdrawal, or protocol deviation. Between-group differences in SPIN scores at 1-month posttreatment and 3-month follow-up will be examined using linear mixed effects models. The model will include group, time, and the group-by-time interaction as fixed effects, baseline SPIN score as a covariate, and a participant-specific random intercept to account for repeated measurements. The data will be automatically generated by Google Forms once participants complete their responses. A *P* value of less than .05 will be used as the level of statistical significance.

#### Secondary Analyses

A similar linear mixed effects modeling approach will be used for secondary outcomes, including depressive symptoms measured by the PHQ-9. Exploratory analyses will examine adherence indicators, including the number of sessions attended, task completion, and homework completion. Where appropriate, adherence may be examined as a predictor or moderator of symptom change. Physiological indicators, including HRV and EDA, will be analyzed descriptively and, where data quality permits, using repeated-measures models to explore within-participant changes across sessions and associations with subjective outcomes.

#### Covariates and Potential Confounding Variables

Selected baseline variables will be included in the final analyses to improve precision. These may include age, gender, baseline social anxiety severity, baseline depressive symptoms, recruitment setting, and prior or concurrent mental health support.

#### Missing Data and Dropout-Related Bias

Linear mixed effects models can accommodate missing outcome data under the missing-at-random assumption. Patterns of missingness and dropout will be examined descriptively by group. If missing data are substantial, multiple imputation or other sensitivity analyses will be conducted to assess the robustness of the findings.

#### Cost Analysis

The cost analysis will encompass the following components: (1) personnel costs, including expenses for the training and recruitment of mental health professionals; (2) VR equipment and software costs, such as VR headsets and software licenses; and (3) materials and supplies (eg, office supplies, printing, and miscellaneous). The total cost will be divided by the average reduction in SPIN scores to calculate the cost per unit reduction in SPIN score.

### Dissemination Plan

Findings will be disseminated through peer-reviewed publications, academic conferences, and knowledge exchange activities with collaborating schools, community organizations, and youth mental health stakeholders. Nontechnical summaries will be prepared for service providers and community partners to support future intervention refinement and implementation planning.

## Results

The pilot study has already generated a ready participant pool, with numerous individuals indicating their willingness to join the VR intervention once it launches. Several partner organizations, including local nongovernmental organizations and middle schools, have also expressed interest in participating in the program. Recruitment for the first cohort is scheduled to begin in March 2026 and continue through December 2026. The study’s design, management, analysis, and reporting are independent of the funder. Demographic features, such as age, gender, and education level, will be reported to provide a comprehensive overview of the participant population. The primary analysis, focusing on social anxiety, will be conducted using an intent-to-treat approach, carefully accounting for missing values. Various visualizations, including trend graphs, charts, flow diagrams, and other illustrative tools, will be used to depict participants’ changes over time. Additionally, subgroup analyses will be included to explore variations within specific segments of the participant population.

Metrics such as intensity and frequency of use will also be reported to provide insights into participants’ engagement with the intervention. Lastly, any significant changes in the software or the delivery of the intervention during the study period will be documented and reported to ensure transparency and contextualize the findings.

## Discussion

### Expected Findings

This study is expected to provide preliminary evidence on the efficacy, feasibility, and acceptability of a therapist-facilitated VR intervention for reducing social anxiety symptoms among youth in Hong Kong. We hypothesize that participants receiving the VR intervention will show greater reductions in SPIN scores than those in the waitlist or delayed-intervention control group at postintervention and at the 3-month follow-up. We also expect to obtain exploratory findings on depressive symptoms, adherence, physiological responses during VR exposure, perceived real-world application, and cost per unit reduction in SPIN scores.

### Comparison With Prior Work

Previous reviews suggest that VR exposure therapy may reduce symptoms of social anxiety disorder and may have effects comparable to other exposure-based approaches in some contexts [26,27]. More broadly, VR therapy has been most extensively studied for anxiety-related conditions and posttraumatic stress disorder, although evidence varies by diagnosis, population, and intervention format [7]. However, the existing literature still contains relatively few trials focused specifically on adolescents and young adults, and many studies differ in therapist involvement, scenario design, follow-up duration, and comparator conditions. Therefore, further randomized trials are needed to clarify how VR-supported interventions may be adapted for youth populations and specific sociocultural contexts.

This study contributes to this body of literature by testing a therapist-facilitated VR intervention that combines structured exposure tasks, therapist preparation and debriefing, culturally relevant social scenarios, physiological monitoring, and AI-supported feedback. Unlike fully self-guided VR interventions, the current intervention retains therapist involvement before and after VR exposure. This design allows therapists to help participants identify feared predictions and safety behaviors, prepare for exposure tasks, review their responses, and plan between-session homework. At the same time, the VR component offers a standardized and immersive environment in which exposure tasks can be repeated and adjusted in difficulty.

### Implications and Future Directions

If the intervention shows preliminary efficacy and acceptability, the findings may inform the design of a larger trial with longer follow-up and a more rigorous comparator condition. Future studies should consider active control groups, such as therapist-led psychoeducation, nonimmersive digital interventions, standard CBT-informed support, or another credible psychological control condition. Such designs would help distinguish the specific contribution of VR from nonspecific effects, such as therapist attention, expectancy, and structured contact. The exploratory cost analysis may also provide initial information on the resources required to deliver the intervention, including therapist time, training, supervision, equipment, software, and administrative costs. However, this trial will estimate the cost per unit reduction in SPIN scores rather than establish full cost-effectiveness in routine clinical or community settings.

### Limitations

Several limitations should be acknowledged. First, given the multimonth research period, participant attrition was a concern. To mitigate this, participants were screened for motivation, supported throughout the study with dedicated staff and reminders, and the VR experience was refined based on pilot feedback to reduce technical issues and enhance engagement. Second, the technological nature of the VR intervention may limit generalizability, as youth more familiar with digital tools may benefit more than older or less tech-savvy individuals. Future research should explore ways to improve accessibility across age groups. Third, the disparity in content and intensity between the VR and control groups may limit interpretability, as symptom improvements could reflect therapist contact or session structure rather than VR-specific effects. Future studies should consider a 3-arm design to better isolate the unique contribution of VR. Fourth, the use of a waitlist control has important methodological limitations. Waitlist control conditions may produce larger apparent treatment effects than more active or usual-care comparators in psychotherapy trials, and they do not control for nonspecific therapeutic factors, such as therapist attention, expectancy, structured contact, or digital engagement [28]. Therefore, the findings will be interpreted as preliminary efficacy compared with delayed intervention rather than as evidence of VR-specific treatment effects. Future trials should include active comparator conditions, such as therapist-led psychoeducation, nonimmersive digital intervention, standard CBT-informed support, or another credible psychological control condition, to better isolate the unique contribution of VR and therapist facilitation.

### Conclusions

In conclusion, this protocol describes an RCT evaluating the preliminary efficacy, feasibility, and acceptability of a therapist-facilitated VR intervention for social anxiety among youth in Hong Kong. By combining immersive VR exposure, therapist preparation and debriefing, physiological monitoring, and lightweight AI-supported prompts, the study will provide initial evidence on whether this intervention is associated with reductions in social anxiety symptoms compared with a waitlist control condition. The study will also generate exploratory findings on depressive symptoms, adherence, perceived real-world application, physiological responses, and cost per unit reduction in SPIN scores. Given the use of a waitlist comparator and a 3-month follow-up period, the findings should be interpreted as preliminary and will not establish VR-specific treatment effects, long-term effectiveness, or broad implementation outcomes. These results may inform the design of future active-controlled trials and longer-term evaluations of VR-supported psychological interventions for youth social anxiety.

## Abbreviations

CBT: cognitive behavioral therapy
CP: control panel
EDA: electrodermal activity
HREC: Human Research Ethics Committee
HRV: heart rate variability
PHQ-9: Patient Health Questionnaire-9
RCT: randomized controlled trial
SPIN: Social Phobia Inventory
SPIRIT: Standard Protocol Items: Recommendations for Interventional Trials
VR: virtual reality

## References

[1] International statistical classification of diseases and related health problems 10th revision (ICD-10) version: 2019. World Health Organization.

[2] (2016). DSM-5 changes: implications for child serious emotional disturbance. https://www.ncbi.nlm.nih.gov/books/NBK519708/pdf/Bookshelf_NBK519708.pdf.

[3] Khalid-Khan S, Santibanez MP, McMicken C, Rynn MA (2007). Social anxiety disorder in children and adolescents: epidemiology, diagnosis, and treatment. Paediatr Drugs.

[4] Erath SA, Flanagan KS, Bierman KL (2007). Social anxiety and peer relations in early adolescence: behavioral and cognitive factors. J Abnorm Child Psychol.

[5] Alden LE, Regambal MJ, Plasencia L, Weeks JW (2014). The Wiley Blackwell Handbook of Social Anxiety Disorder.

[6] Beesdo-Baum K, Knappe S, Asselmann E (2015). The 'Early Developmental Stages of Psychopathology (EDSP) study': a 20-year review of methods and findings. Soc Psychiatry Psychiatr Epidemiol.

[7] Emmelkamp PMG, Meyerbröker K (2021). Virtual reality therapy in mental health. Annu Rev Clin Psychol.

[8] Carl E, Stein AT, Levihn-Coon A (2019). Virtual reality exposure therapy for anxiety and related disorders: a meta-analysis of randomized controlled trials. J Anxiety Disord.

[9] Kothgassner OD, Felnhofer A (2021). Lack of research on efficacy of virtual reality exposure therapy (VRET) for anxiety disorders in children and adolescents: a systematic review. Neuropsychiatry.

[10] Morina N, Ijntema H, Meyerbröker K, Emmelkamp PMG (2015). Can virtual reality exposure therapy gains be generalized to real-life? A meta-analysis of studies applying behavioral assessments. Behav Res Ther.

[11] Leigh E, Clark DM (2018). Understanding social anxiety disorder in adolescents and improving treatment outcomes: applying the cognitive model of Clark and Wells (1995). Clin Child Fam Psychol Rev.

[12] Clark DM, Ehlers A, Hackmann A (2006). Cognitive therapy versus exposure and applied relaxation in social phobia: a randomized controlled trial. J Consult Clin Psychol.

[13] Botella C, Fernández-Álvarez J, Guillén V, García-Palacios A, Baños R (2017). Recent progress in virtual reality exposure therapy for phobias: a systematic review. Curr Psychiatry Rep.

[14] Sinyor M, Hawton K, Appleby L (2023). The coming global economic downturn and suicide: a call to action. Nat Mental Health.

[15] Olusanya BO, Davis AC, Hadders-Algra M, Wright SM (2023). Global investments to optimise the health and wellbeing of children with disabilities: a call to action. Lancet.

[16] Freeman D, Lister R, Waite F (2023). Automated virtual reality cognitive therapy versus virtual reality mental relaxation therapy for the treatment of persistent persecutory delusions in patients with psychosis (THRIVE): a parallel-group, single-blind, randomised controlled trial in England with mediation analyses. Lancet Psychiatry.

[17] Garcia-Lopez LJ, Sáez-Castillo AJ, Beidel D, La Greca AM (2015). Brief measures to screen for social anxiety in adolescents. J Dev Behav Pediatr.

[18] Furmark T (2000). Social phobia. From epidemiology to brain function [Dissertation]. https://swepub.kb.se/bib/swepub%3Aoai%3ADiVA.org%3Auu-546?language=en&tab2=abs.

[19] Heimberg RG, Liebowitz MR, Hope DA, Schneier FR (1995). Social Phobia: Diagnosis, Assessment, and Treatment.

[20] McManus F, Sacadura C, Clark DM (2008). Why social anxiety persists: an experimental investigation of the role of safety behaviours as a maintaining factor. J Behav Ther Exp Psychiatry.

[21] Yuen ASY, Mak WWS (2021). The effects of immersive virtual reality in reducing public stigma of mental illness in the university population of Hong Kong: randomized controlled trial. J Med Internet Res.

[22] Connor KM, Davidson JR, Churchill LE, Sherwood A, Foa E, Weisler RH (2000). Psychometric properties of the Social Phobia Inventory (SPIN). New self-rating scale. Br J Psychiatry.

[23] Antony MM, Coons MJ, McCabe RE, Ashbaugh A, Swinson RP (2006). Psychometric properties of the social phobia inventory: further evaluation. Behav Res Ther.

[24] Chen YS (2009). Screening for social phobia: psychometric evaluation of Chinese Social Phobia Inventory (SPIN). J Chin Med Assoc.

[25] Wang W, Bian Q, Zhao Y (2014). Reliability and validity of the Chinese version of the Patient Health Questionnaire (PHQ-9) in the general population. Gen Hosp Psychiatry.

[26] Horigome T, Kurokawa S, Sawada K (2020). Virtual reality exposure therapy for social anxiety disorder: a systematic review and meta-analysis. Psychol Med.

[27] Tan YL, Chang VYX, Ang WHD, Ang WW, Lau Y (2025). Virtual reality exposure therapy for social anxiety disorders: a meta-analysis and meta-regression of randomized controlled trials. Anxiety Stress Coping.

[28] Furukawa TA, Noma H, Caldwell DM (2014). Waiting list may be a nocebo condition in psychotherapy trials: a contribution from network meta-analysis. Acta Psychiatr Scand.

